# Supplementary material for: Compositional Changes in the Vaginal Bacterial Microbiome of Healthy Pregnant Women across the Three Gestational Trimesters in Ismailia, Egypt
Source: Microorganisms. 2023 Jan 5;11(1):139. doi: 10.3390/microorganisms11010139 (PMC9862816; doi:10.3390/microorganisms11010139)
Supplement: Supplementary file 1 [file microorganisms-11-00139-s001.zip › R_scripts_modified_21-11-2022.pdf]

# R script of collective taxonomy chart of relative abundances of taxa at phylum, genus and species levels using facet wrap package:

```
# R version 4.2.0
#load packages
library(tidyverse)
library(ggplot2)
library(readxl)


#import phyla data
Phyla <- read_excel("File path")
View(Phyla)


# Ploting Phyla with ggplot
Phyla_plot <- ggplot(Phyla, aes(x = Pregnancy_status, y = Relative_abundance, fill = Phylum)) +
geom_bar(position = position_dodge(),
stat="identity") +
facet_wrap(~Phylum) +
 theme(axis.text.x =
element_text(colour="gray0",size=8,angle=90,hjust=.5,vjust=.5,face="plain"),
axis.text.y = element_text(colour="gray0",size=10,angle=0,hjust=1,vjust=0,face="plain"),
axis.title.x = element_text(colour="gray0",size=12,angle=0,hjust=.5,vjust=0,face="bold"),
axis.title.y = element_text(colour="gray0",size=12,angle=90,hjust=.5,vjust=.5,face="bold")) +
theme(legend.text=element_text(size=8)) +
theme(legend.title=element_text(size=10, face = "bold")) +
ggtitle("Relative abundance of taxa at phylum level")+
theme(plot.title = element_text(hjust = 0.5, size = 12, face = "bold"))
Phyla_plot
P1 <- Phyla_plot
P1

#import genus data
genera <- read_excel("File path")
View(genera)
#genera plot with ggplot
Genera_plot <- ggplot(genera, aes(x = Pregnancy_status, y = Relative_abundance, fill = Genus)) +
geom_bar(position = position_dodge(),
stat="identity") +
facet_wrap(~Genus) +
theme(axis.text.x = element_text(colour="gray0",size=8,angle=90,hjust=.5,vjust=.5,face="plain"),
```

```
axis.text.y = element_text(colour="gray0",size=10,angle=0,hjust=1,vjust=0,face="plain"),
axis.title.x = element_text(colour="gray0",size=12,angle=0,hjust=.5,vjust=0,face="bold"),
axis.title.y = element_text(colour="gray0",size=12,angle=90,hjust=.5,vjust=.5,face="bold")) +
theme(legend.text=element_text(size=8)) +
theme(legend.title=element_text(size=10, face = "bold")) +
ggtitle("Relative abundance of taxa at genus level")+
theme(plot.title = element_text(hjust = 0.5, size = 12, face = "bold"))
Genera_plot
P2 <- Genera_plot
P2

#import species belong lactobacillus
species_lactobacillus <- read_excel("File Path")
View(species_lactobacillus)
# plot species belong lactobacillus with ggplot

species_lactobacillus_plot <-ggplot(species_lactobacillus, aes(x = Pregnancy_status, y =
Relative_abundance, fill = Species)) + geom_bar(position = position_dodge(),

stat="identity") +
  facet_wrap(~Species) +
  theme(axis.text.x =
element_text(colour="gray0",size=8,angle=90,hjust=.5,vjust=.5,face="plain"),
        axis.text.y = element_text(colour="gray0",size=10,angle=0,hjust=1,vjust=0,face="plain"),
        axis.title.x = element_text(colour="gray0",size=12,angle=0,hjust=.5,vjust=0,face="bold"),
        axis.title.y = element_text(colour="gray0",size=12,angle=90,hjust=.5,vjust=.5,face="bold")) +
  theme(legend.text=element_text(size=8)) +
  theme(legend.title=element_text(size=10, face = "bold")) +
  ggtitle("Relative abundance of taxa at species level belong to Lactobacillus")+
  theme(plot.title = element_text(hjust = 0, size = 12, face = "bold"))

final_species_lactobacillus_plot <- species_lactobacillus_plot + ylim(0, 1)

final_species_lactobacillus_plot

P3 <- final_species_lactobacillus_plot

P3
```

```
#import data not belong lactobacillus:
species_nonlactobacillus <- read_excel("File Path")
View(species_nonlactobacillus)

#Plot species_nonlactobacillus with ggplot
species_nonlactobacillus_plot <-ggplot(species_nonlactobacillus, aes(x = Pregnancy_status, y =
Relative_abundance, fill = Species)) + geom_bar(position = position_dodge(),
                                                              stat="identity")
+
facet_wrap(~Species) +
theme(axis.text.x = element_text(colour="gray0",size=8,angle=90,hjust=.5,vjust=.5,face="plain"),
axis.text.y = element_text(colour="gray0",size=10,angle=0,hjust=1,vjust=0,face="plain"),
axis.title.x = element_text(colour="gray0",size=12,angle=0,hjust=.5,vjust=0,face="bold"),
axis.title.y = element_text(colour="gray0",size=12,angle=90,hjust=.5,vjust=.5,face="bold")) +
theme(legend.text=element_text(size=8)) +
theme(legend.title=element_text(size=10, face = "bold")) +
ggtitle("Relative abundance of taxa at species level not belong to Lactobacillus")+
theme(plot.title = element_text(hjust = 0, size = 12, face = "bold"))

final_species_nonlactobacillus_plot <- species_nonlactobacillus_plot + ylim(0, 1)

final_species_nonlactobacillus_plot

P4 <- final_species_nonlactobacillus_plot


P4

#combine plots
#install.packages("cowplot")
library(cowplot)
Final_image <- plot_grid(P1, P2, P3, P4, nrow = 2, ncol = 2, rel_widths = c(1, 1), labels = c("(A)",
"(B)", "(C)", "(D)"))
Final_image
ggsave("taxonomy.tiff", units="in", width=14, height=8, dpi=300)
```

#R script for collective alpha diversity chart:

```
#Load packages
library(ggstatsplot)
library(readxl)
library(ggplot2)
```

```
library(cowplot)
import data
library(readxl)

#Import alpha diversity excel tables generated by MicrobiomeAnalyst
observed_alpha_diversity_index <- read_excel("File Path")
View(observed_alpha_diversity_index)
Chao1_alpha_diversity_index <- read_excel("File Path")
View(Chao1_alpha_diversity_index)
Shannon_alpha_diversity_index <- read_excel("File Path")
View(Shannon_alpha_diversity_index)
Simpson_alpha_diversity_index <- read_excel("File Path")
View(Simpson_alpha_diversity_index)


#Plot observed_alpha_diversity_index
Observed <- ggbetweenstats(observed_alpha_diversity_index, x = pregnancy_status, y = observed, type = "nonparametric", pairwise.display = "s", xlab = "Pregnancy_status", ylab = "Alpha_diversity: Observed", centrality.label.args = list(size  = 3), centrality.point.args = list(size = 5, color = "#000000"), point.args = list(position = ggplot2::position_jitterdodge(dodge.width = 0.6), alpha = 1, size = 4, stroke = 0), ggtheme = ggplot2::theme(axis.text =element_text(color = "black", size = 10), axis.title = element_text(face = "bold", colour = "black", size = 12), panel.border = element_rect  (colour = "black", size = 1, fill = NA), panel.background = element_rect(fill = "gray97", color = "gray97")))

Observed

#To change colours

p1 <- Observed + ggplot2::scale_fill_manual(values = c("#006400", "#8B0000", "#0000CD", "#EEC900"), aesthetics = c("colour", "fill"))
p1

#Plot Chao1_alpha-diversity-index
Chao1 <- ggbetweenstats(Chao1_alpha_diversity_index, x = pregnancy_status, y = chao1, type = "nonparametric", pairwise.display = "s", xlab = "Pregnancy_status", ylab = "Alpha_diversity: Chao1", centrality.label.args = list(size  = 3), centrality.point.args = list(size = 5, color = "#000000"), point.args = list(position = ggplot2::position_jitterdodge(dodge.width = 0.6), alpha = 1, size = 4, stroke = 0),
ggtheme = ggplot2::theme(axis.text =element_text(color = "black", size = 10), axis.title = element_text(face = "bold", colour = "black", size = 12), panel.border = element_rect  (colour = "black", size = 1, fill = NA), panel.background = element_rect(fill = "gray97", color = "gray97")))
```

```
p2 <- Chao1 + ggplot2::scale_fill_manual(values = c("#006400", "#8B0000", "#0000CD",
"#EEC900"), aesthetics = c("colour", "fill"))

p2

#Plot Shannon_alpha_diversity_index
Shanon <- ggbetweenstats(Shannon_alpha_diversity_index, x = pregnancy_status, y = shannon,
type = "nonparametric", pairwise.display = "s", xlab = "Pregnancy_status", ylab =
"Alpha_diversity: Shannon", centrality.label.args = list(size  = 3), centrality.point.args = list(size =
5, color = "#000000"), point.args = list(position = ggplot2::position_jitterdodge(dodge.width =
0.6), alpha = 1, size = 4, stroke = 0),
ggtheme = ggplot2::theme(axis.text =element_text(color = "black", size = 10), axis.title =
element_text(face = "bold", colour = "black", size = 12), panel.border = element_rect  (colour =
"black", size = 1, fill = NA), panel.background = element_rect(fill = "gray97", color = "gray97")))

p3 <- Shanon + ggplot2::scale_fill_manual(values = c("#006400", "#8B0000", "#0000CD",
"#EEC900"), aesthetics = c("colour", "fill"))

p3

#Plot Simpson_alpha_diversity_index
Simpson <- ggbetweenstats(Simpson_alpha_diversity_index, x = pregnancy_status, y = simpson,
type = "nonparametric", pairwise.display = "s", xlab = "Pregnancy_status", ylab =
"Alpha_diversity: Simpson", centrality.label.args = list(size  = 3), centrality.point.args = list(size =
5, color = "#000000"), point.args = list(position = ggplot2::position_jitterdodge(dodge.width =
0.6), alpha = 1, size = 4, stroke = 0), ggtheme = ggplot2::theme(axis.text =element_text(color =
"black", size = 10), axis.title = element_text(face = "bold", colour = "black", size = 12),
panel.border = element_rect  (colour = "black", size = 1, fill = NA), panel.background =
element_rect(fill = "gray97", color = "gray97")))
p4 <- pf + ggplot2::scale_fill_manual(values = c("#006400", "#8B0000", "#0000CD", "#EEC900"),
aesthetics = c("colour", "fill"))

p4

#combine plots
install.packages("cowplot")
install.packages("ggpubr ")

library(cowplot)
library(ggpubr)


final_image <- plot_grid(p1, p2, p3, p4, nrow = 2, ncol = 2, rel_widths = c(1, 1), labels = c("(A)",
"(B)", "(C)", "(D)"))
```

final_image

ggsave("Alpha-diversity.tiff", units="in", width=12, height=6, dpi=300)

#R script for Pielou evenness alpha diversity chart:

#import data
library(readxl)
pielou_eveness_alpha_diversity <- read_excel("File Path")
View(pielou_eveness_alpha_diversity)

#load package
library(ggstatsplot)
library(tidyverse)
library(ggplot2)

pielou_eveness_plot <- ggbetweenstats(pielou_eveness_alpha_diversity, x = pregnancy_status, y = pielou_evenness, type = "nonparametric",

pairwise.display = "all", xlab = "Pregnancy_status", ylab = "Alpha_diversity: Pielou_evenness",

centrality.label.args = list(size = 4), centrality.point.args = list(size = 5, color = "#000000"),

point.args = list(position = ggplot2::position_jitterdodge(dodge.width = 0.6), alpha

= 1, size = 4, stroke = 0),

ggtheme = ggplot2::theme(axis.text =element_text(color = "black", size = 10),

axis.title = element_text(face = "bold", colour = "black", size = 12), panel.border = element_rect (colour = "black", size = 1, fill = NA),

panel.background = element_rect(fill = "gray97", color = "gray97")))

pielou_eveness_plot

#To change colours

pielou_eveness_plot_modified <- pielou_eveness_plot + ggplot2::scale_fill_manual(values = c("#006400", "#8B0000", "#0000CD", "#EEC900"), aesthetics = c("colour", "fill"))

pielou_eveness_plot_modified

ggsave("pielou_evenness.tiff", units="in", width=6, height=6, dpi=300)

#R script for correlation at phylum level:

```
#import data:
library(readxl)
correlation_phylum <- read_excel("File Path")
View(correlation_phylum)

#Calculate correlation
library("Hmisc")
correlation_result <- rcorr(as.matrix(correlation_phylum), type = "spearman")
correlation_result

#To calculate r and p values for n samples and save in tsv file
#Extract p value
correlation_p_value <- correlation_result $P
correlation_p_value

#Round p value
p_value <- round(correlation_p_value, 2)

#Extract r value
correlation_r_value <- correlation_result$r
correlation_r_value

#Round r value
r_value <- round(correlation_r_value, 2)


#To make tsv output file
sink("correlation_phyla.tsv")
correlation_result <- rcorr(as.matrix(correlation_phylum), type = "spearman")
correlation_result

correlation_p_value <- correlation_result $P
correlation_p_value
round(correlation_p_value, 2)
correlation_r_value <- correlation_result $r
correlation_r_value
round(correlation_r_value, 2)
correlation_n_phyla <- correlation_result$n
```

```
correlation_n_phyla
sink()

#Plot correlation
library("colorRamps")
library(heatmaply)
library(viridis)
library(plotly)
library(shiny)


#Pallette for correlation plot
my_palette2 <- colorRampPalette(c("blue4", "lightcyan1", "darkred"))(n = 299)

p1 <- heatmaply_cor(r_value
,limits = c(-0.5, 1), colors = my_palette2, column_text_angle = -90, fontsize_row = 14,
fontsize_col = 14, plot_method = "plotly")

p1


#import data for comparitive correlation phyla
library(readxl)
Comparative_correlation_phylum <- read_excel("File Path")
View(Comparative_correlation_phylum)
#load packages for pairwise graphical comparison of several distributions
library(PerformanceAnalytics)
library(tidyverse)
library(GGally)

#Plot comparative correlation
p2 <- ggpairs(Comparative_correlation_phylum, columns = 1:7,
      aes(colour = Pregnancy_Trimester),
      lower = list(continuous = "smooth"),
      upper = list(continuous = wrap("cor", method = "spearman", size = 3.5)))
p2
ggsave("Comparative_correlation_phylum.tiff", units="in", width=10, height=7, dpi=300)
```

R script for correlation at genus level:

```
#import data:
library(readxl)
correlation_genus <- read_excel("File Path")
View(correlation_genus)

#Calculate correlation
library("Hmisc")
correlation_result_genera <- rcorr(as.matrix(correlation_genus), type = "spearman")
correlation_result_genera

#To calculate r and p values for n samples and save in tsv file
#Extract p value
correlation_p_value_genera <- correlation_result_genera $P
correlation_p_value_genera

#Round p value
p_value_genera <- round(correlation_p_value_genera, 2)

#Extract r value
correlation_r_value_genera <- correlation_result_genera$r
correlation_r_value_genera

#Round r value
r_value_genera <- round(correlation_r_value_genera, 2)


#To make tsv output file
sink("correlation_genera.tsv")
correlation_result_genera <- rcorr(as.matrix(correlation_genus), type = "spearman")
correlation_result_genera

correlation_p_value_genera <- correlation_result_genera $P
correlation_p_value_genera
round(correlation_p_value_genera, 2)
correlation_r_value_genera <- correlation_result_genera $r
correlation_r_value_genera
round(correlation_r_value_genera, 2)
correlation_n_genera <- correlation_result_genera$n
correlation_n_genera
```

```
sink()


#Plot correlation
library("colorRamps")
library(heatmaply)
library(viridis)
library(plotly)
library(shiny)


#Pallette for correlation plot
my_palette2 <- colorRampPalette(c("blue4", "lightcyan1", "darkred"))(n = 299)

p3 <- heatmaply_cor(r_value_genera
,limits = c(-0.5, 1), colors = my_palette2, column_text_angle = -90, fontsize_row = 14,
fontsize_col = 14, plot_method = "plotly")

p3

#to close any opened plot

dev.off()

#import data for comparitive correlation genera
library(readxl)
Comparative_correlation_genus <- read_excel("File Path")
View(Comparative_correlation_genus)
#load packages for pairwise graphical comparison of several distributions
library(PerformanceAnalytics)
library(tidyverse)
library(GGally)


#Plot comparative correlation
p4 <- ggpairs(Comparative_correlation_genus, columns = 1:10,
      aes(colour = Pregnancy_Trimester),
      lower = list(continuous = "smooth"),
      upper = list(continuous = wrap("cor", method = "spearman", size = 3.5)))
p4
#to change font size
final_figure <- p4 + theme(text = element_text(size = 8))
final_figure
#then export as PNG image.
```

#R script for correlation at species level:

```
#import data:
library(readxl)
correlation_species <- read_excel("File Path")
View(correlation_species)

#Calculate correlation
library("Hmisc")
correlation_result_species <- rcorr(as.matrix(correlation_species), type = "spearman")
correlation_result_species

#To calculate r and p values for n samples and save in tsv file
#Extract p value
correlation_p_value_species <- correlation_result_species $P
correlation_p_value_species

#Round p value
p_value_species <- round(correlation_p_value_species, 2)

#Extract r value
correlation_r_value_species <- correlation_result_species$r
correlation_r_value_species

#Round r value
r_value_species <- round(correlation_r_value_species, 2)


#To make tsv output file
sink("correlation_species.tsv")
correlation_result_species <- rcorr(as.matrix(correlation_species), type = "spearman")
correlation_result_species

correlation_p_value_species <- correlation_result_species $P
correlation_p_value_species
round(correlation_p_value_species, 2)
correlation_r_value_species <- correlation_result_species $r
correlation_r_value_species
round(correlation_r_value_species, 2)
correlation_n_species <- correlation_result_species$n
correlation_n_species
```

```
sink()


#Plot correlation
library("colorRamps")
library(heatmaply)
library(viridis)
library(plotly)
library(shiny)


#Pallette for correlation plot
my_palette2 <- colorRampPalette(c("blue4", "lightcyan1", "darkred"))(n = 299)

p5 <- heatmaply_cor(r_value_species
,limits = c(-0.5, 1), colors = my_palette2, column_text_angle = -90, fontsize_row = 14,
fontsize_col = 14, plot_method = "plotly")

p5

#to close any opened plot

dev.off()

#import data for comparitive correlation of Lactobacillus species
library(readxl)
Comparative_correlation_species_lactobacillus <- read_excel("File Path")
View(Comparative_correlation_species_lactobacillus)
#load packages for pairwise graphical comparison of several distributions
library(PerformanceAnalytics)
library(tidyverse)
library(GGally)


#Plot comparative correlation
p6 <- ggpairs(Comparative_correlation_species_lactobacillus, columns = 1:9,
       aes(colour = Pregnancy_Trimester),
       lower = list(continuous = "smooth"),
       upper = list(continuous = wrap("cor", method = "spearman", size = 3.5)))
p6
#to change font size
final_figure <- p6 + theme(text = element_text(size = 8))
final_figure
#then export as PNG image
```

```
#to know directory where output files were saved
getwd()

#import data for comparitive correlation of nonLactobacillus species
library(readxl)
Comparative_correlation_species_nonlactobacillus <- read_excel("File Path")
View(Comparative_correlation_species_nonlactobacillus)
#load packages for pairwise graphical comparison of several distributions
library(PerformanceAnalytics)
library(tidyverse)
library(GGally)

#Plot comparative correlation
p7 <- ggpairs(Comparative_correlation_species_nonlactobacillus, columns = 1:9,
       aes(colour = Pregnancy_Trimester),
       lower = list(continuous = "smooth"),
       upper = list(continuous = wrap("cor", method = "spearman", size = 3.5)))
p7
#to change font size
final_figure <- p7 + theme(text = element_text(size = 8))
final_figure
#then export as PNG image
```

#R script for CST bar chart using facet wrap:

#First we run the following script in Linux terminal (which is available on the Valencia github page):

The script is run as follows:

```
python3 /path/to/Valencia.py -ref /path/to/CST_profiles_012920.csv -i
/path/to/test_dataset.csv -o /path/to/test_out -p /path/to/test

#import data
library(readxl)
cst <- read_excel("File Path")
View(cst)


load library
library(tidyverse)
```

```
library(ggplot2)

#Plot cst
ggplot(cst, aes(x = pregnancy_status, y = Total_sample_No., fill = CST)) +
 geom_bar(stat = "identity", position = "dodge") +
 facet_wrap(~CST) +
 theme(axis.text.x =
element_text(colour="gray0",size=8,angle=90,hjust=.5,vjust=.5,face="plain"),
axis.text.y = element_text(colour="gray0",size=10,angle=0,hjust=1,vjust=0,face="plain"),
axis.title.x = element_text(colour="gray0",size=12,angle=0,hjust=.5,vjust=0,face="bold"),
axis.title.y = element_text(colour="gray0",size=12,angle=90,hjust=.5,vjust=.5,face="bold")) +
theme(legend.text=element_text(size=8)) +
theme(legend.title=element_text(size=10, face = "bold")) +
ggtitle("VALENCIA CST classification")+
theme(plot.title = element_text(hjust = 0.5, size = 12, face = "bold"))

ggsave("CST_VALENCIA.tiff", units="in", width=10, height=6, dpi=300)
```
