# Supplementary figures and images for: Compositional Changes in the Vaginal Bacterial Microbiome of Healthy Pregnant Women across the Three Gestational Trimesters in Ismailia, Egypt
Source: Microorganisms. 2023 Jan 5;11(1):139. doi: 10.3390/microorganisms11010139 (PMC9862816; doi:10.3390/microorganisms11010139)

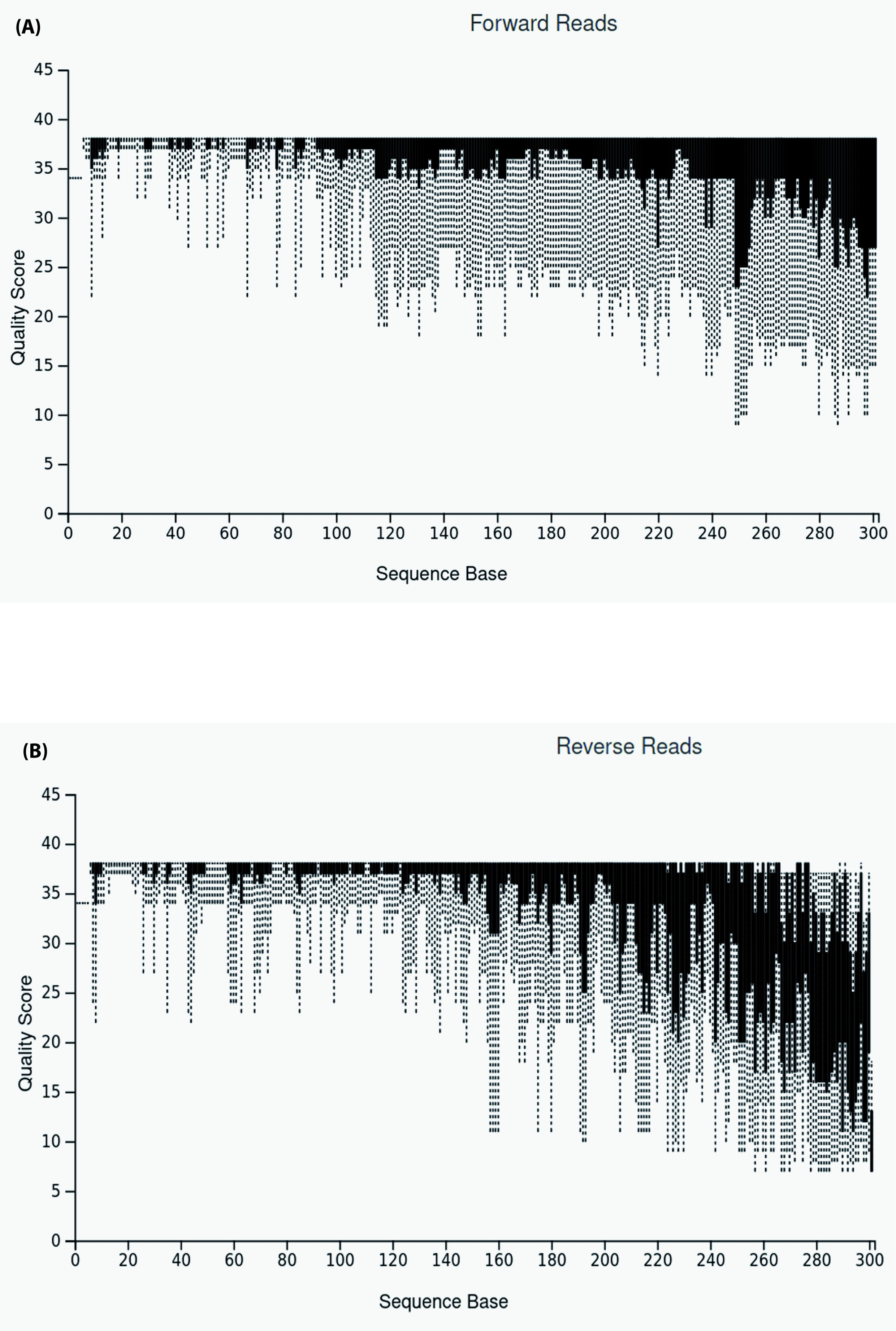

Supplement: Supplementary file 1 [file microorganisms-11-00139-s001.zip › Supplementary_Figure_S1_positional_quality_plots.tif]

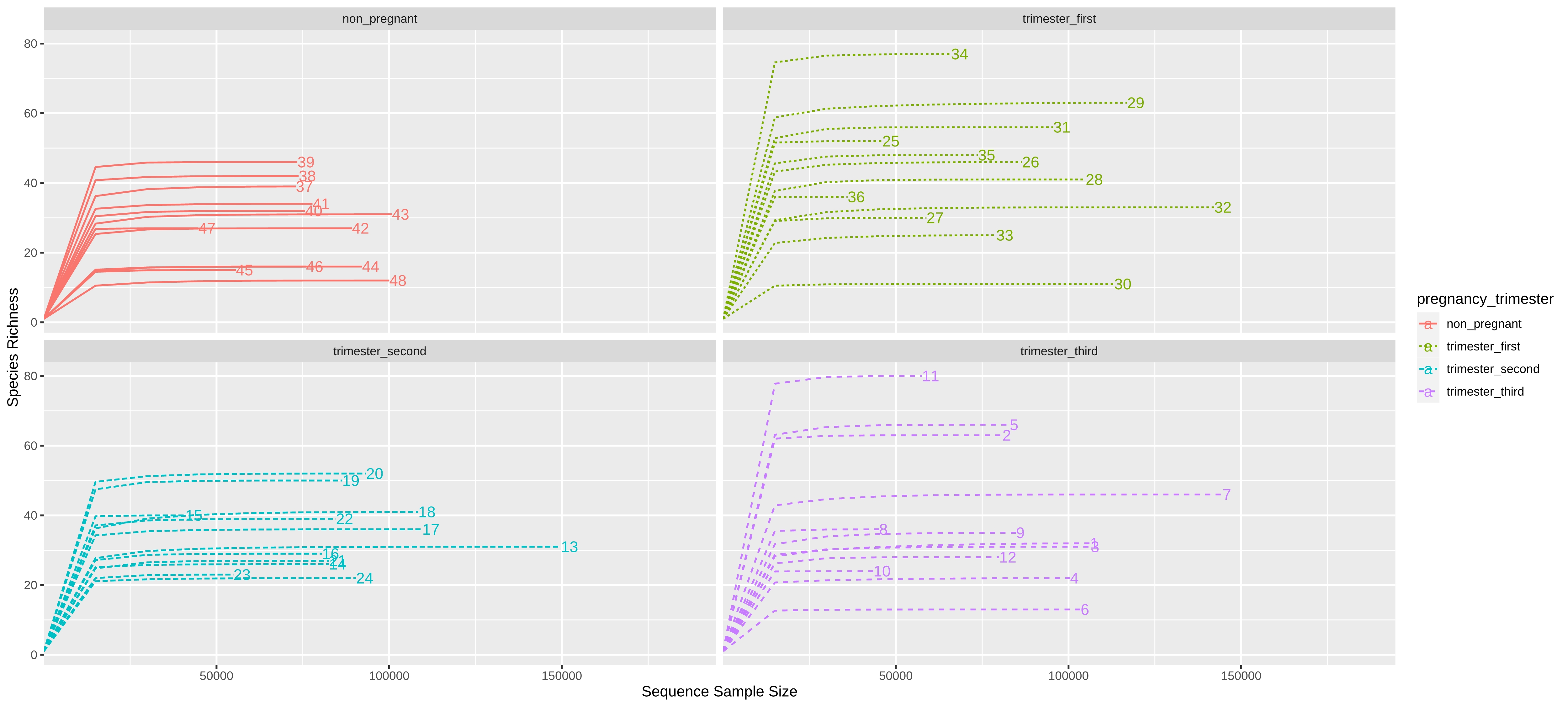

Supplement: Supplementary file 1 [file microorganisms-11-00139-s001.zip › Supplementary_Figure_S2_rarefaction_curve.jpeg]

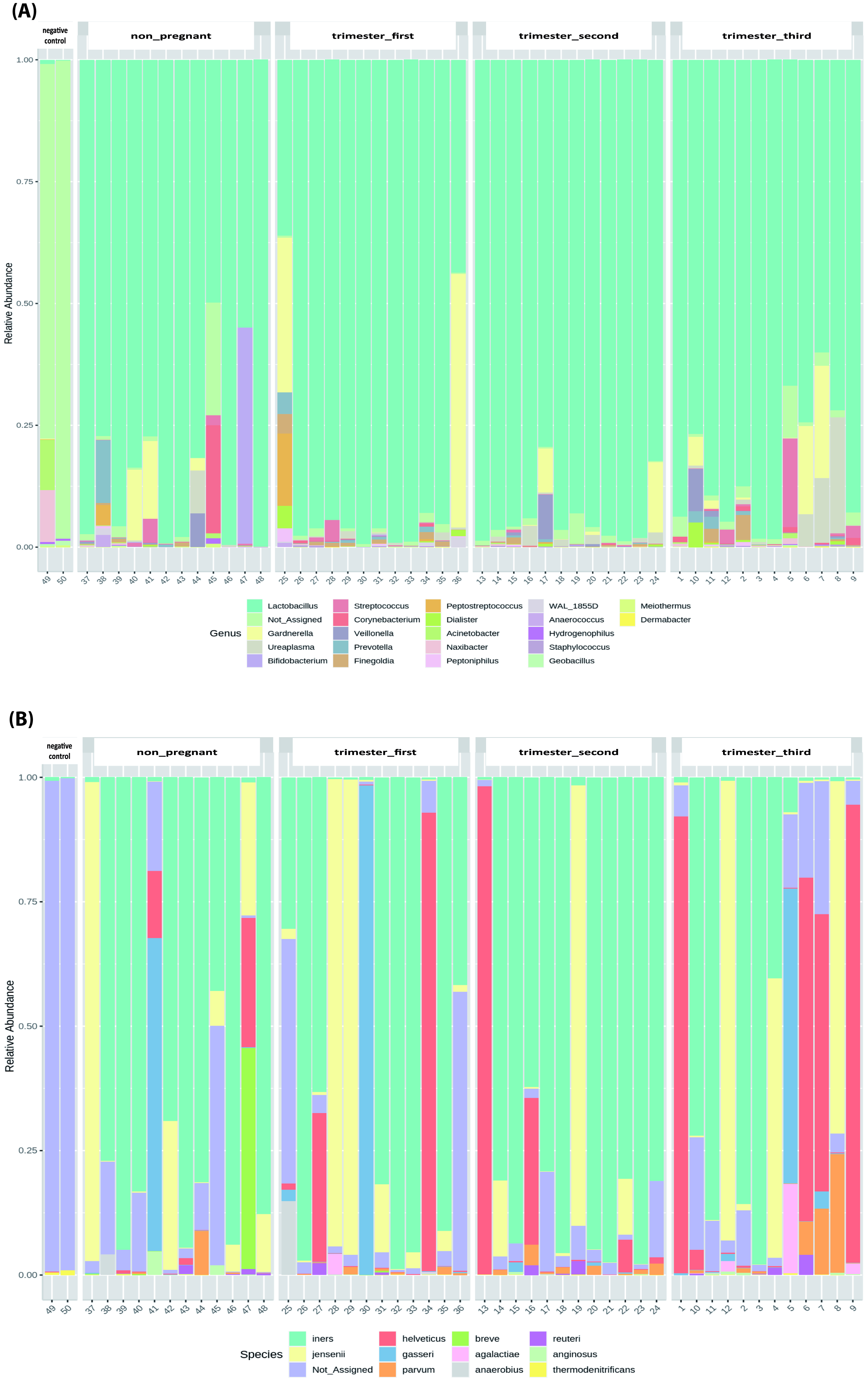

Supplement: Supplementary file 1 [file microorganisms-11-00139-s001.zip › Supplementary_Figure_S3_Stacked_bar_charts_of_individual_samples_modified_5-1-2023.tif]
